# Our Three Musketeers: A Case Series of NLRP-3 Associated Cryopyrinopathies

**DOI:** 10.31138/mjr.240624.eyr

**Published:** 2025-06-30

**Authors:** Sumanth Madan, Spoorthy Raj, Sudeep Rath, Joslyn M. Thattil, Ashvin Pillai, Mithun CB, Suma Balan

**Affiliations:** Department of Clinical Immunology and Rheumatology, Amrita Institute of Medical Sciences, Kochi, India

**Keywords:** NLRP3, autoinflammatory diseases, sacroiliitis, cryopyrinopathy

## Abstract

Autoinflammatory diseases affecting the NLRP3 gene are rare autosomal dominant disorders presenting with episodic organ limited and systemic inflammation. We report three patients with cryopyrinopathies. Our first case is a 4-year-old boy with a history of periodic fever, failure to thrive, and raised intracranial pressure. The second case is a 6-year-old boy with similar complaints, also with bilateral uveitis. The third is a 24-year-old gentleman with periodic fever and early hearing loss, also with a novel presentation of sacroiliitis. Our case series demonstrates that there should be a low clinical threshold indicating genetic testing in any child who displays features of autoinflammation in combination with an urticarial rash, musculoskeletal manifestations, hearing loss, and chronic aseptic meningitis with macrocephaly. Furthermore, despite anakinra being a cornerstone in treating NLRP-3 AID, there is an unmet clinical need to provide access to alternatives such as colchicine and thalidomide in resource-limited settings.

## INTRODUCTION

*NLRP* 3-associated autoinflammatory diseases (*NLRP*3-AID) are a rare group of disorders consisting of three clinically overlapping yet unique syndromes with varying severity. It is clinically defined by systemic, cutaneous, musculoskeletal, and central nervous system inflammation episodes.^1^

The *CIAS1* gene is also synonymous with *NLRP3,* which encodes the cryopyrin protein. This is a critical component of the inflammasome, a complex within the cell responsible for producing interleukin (IL)-1.^2^ Gain-of-function mutations in *NLRP3* often result in excessive production of interleukins, resulting in overall systemic inflammation and a consequent spectrum of presentations.^3,4^

This spectrum encompasses three autosomal dominant diseases caused by heterozygous gain of function mutations in the *NLRP3* gene, including familial cold autoinflammatory syndrome (FCAS, mild phenotype), Muckle-Wells syndrome (MWS, Intermediate phenotype), and in its most severe form; neonatal-onset multisystem inflammatory disease (NOMID). This persistent systemic and CNS inflammation culminates in papilledema, aseptic meningitis, sensorineural hearing loss, cerebral atrophy, and secondary AA amyloidosis. The purpose of this case review is to present three cases of *NLRP*-3 AID displaying the varying severity and challenges faced in the diagnosis and management.

## CASE DESCRIPTIONS

### P1

**Figure 1.**
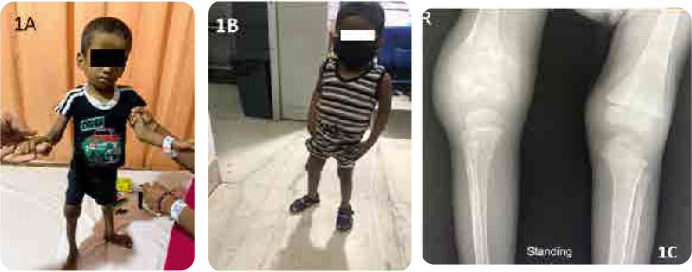
Patient 1. **A.** Patient 1 Emaciated, Frontal bossing, bony swelling of the right knee. **B.** Significant weight gain with treatment. **C.** Plain X-Ray of bilateral knees demonstrating persistent right joint effusion and swelling with soft tissue involvement. Abnormal widening and cupping of distal metaphyses, enlarged epiphyses with trabeculations.

A 4-year-old boy with a history of recurrent infections, persistent diarrhoea, periodic fever, and failure to thrive came with urticarial evanescent rashes and right knee swelling with difficulty in weight-bearing and poor appetite. During his neonatal period, he was evaluated for recurrent episodes of systemic sterile inflammation without an identifiable infectious trigger. A provisional diagnosis of immunodeficiency (leukocyte adhesion defect) was considered due to the history of delayed umbilical cord separation and exuberant neutrophilic leucocytosis. However, the diagnosis was not established. On examination, the child was irritable and had frontal bossing and an open anterior fontanelle. A musculoskeletal examination showed a bony swelling of the right knee, and the child was unable to bear weight (**Figure 1A–C**).

A multidisciplinary team, including paediatric neurology, otorhinology, and ophthalmology in conjunction with neuroimaging, determined papilledema and moderate sensorineural hearing loss with no evidence of cerebral atrophy. Because of the above findings, a revised diagnosis of neonatal-onset multisystem inflammatory disease was considered, and whole exome sequencing was performed. The child was started on oral glucocorticoids at 2mg/kg/day, thalidomide and acetazolamide 250 mg/day while we awaited access to anakinra.

Following discharge, he was eventually started on anakinra (33.3 mg subcutaneous) daily and has since made a dramatic recovery in all clinical domains except for hearing loss and persistence of knee swelling **(Table 1, Figure 1C).**

**Table 1.** Improvement in domains of organ involvement and chronic inflammation (P1).

**Lab investigations**	**Pre-treatment**	**Latest follow up**
**Weight (kgs)**	9	17.5
**Haemoglobin (g/dl)**	4	11
**TLC (/uL)**	19920	10850
**Platelets (K/uL)**	508000	363000
**ESR (mm/hr)**	140	12
**CRP (mg/dL)**	107	0.5
**Fundus evaluation**	Papilledema	No active papilledema

### P2

**Figure 2.**
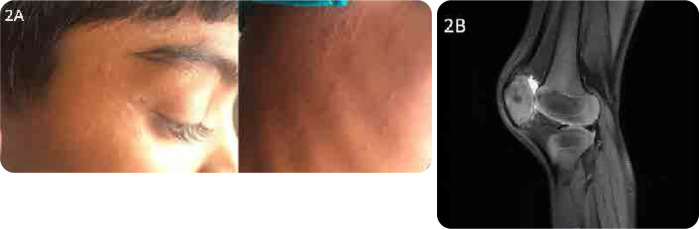
Patient 2. **A.** Shows urticarial erythematous papular rash with wheal upon presentation. **B.** MRI of the knee: enlarged patellae with irregular outlines suggestive of persistent NLRP3-associated arthropathy/hyperostosis.

An 8-year-old boy presented to us with recurrent episodes of fever associated with urticarial rash at the age of 3 (**Figure 2A**). Each episode of moderate to high-grade fever is associated with bilateral knee arthritis lasting for 2–3 days. He had a similar history of urticarial rash in the neonatal period. His evaluation included routine blood tests, genetic testing, ophthalmic examination, ENT assessment, and neuroimaging. Ophthalmological examination showed features of uveitis. As a bridge therapy, he was started on alternate-day lenalidomide (5mg) and low-dose prednisolone at the maximum tolerated dose. However, he only had a partial response, manifested by intermittent flares, albeit less severe than previous flares. Despite this treatment, he progressed to have hyperostosis of the knee on follow-up (**Figure 2B**). He has since been started on Anakinra and has been doing very well with no further flares.

### P3

**Figure 3.**
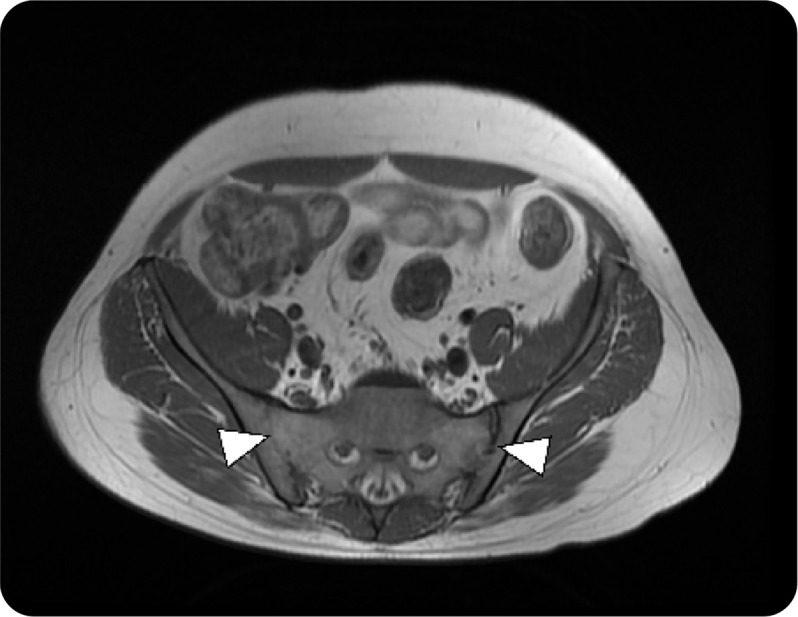
**(above).** T1W MRI of P3 demonstrating bilateral ankylosis (White arrows).

**Figure 4.**
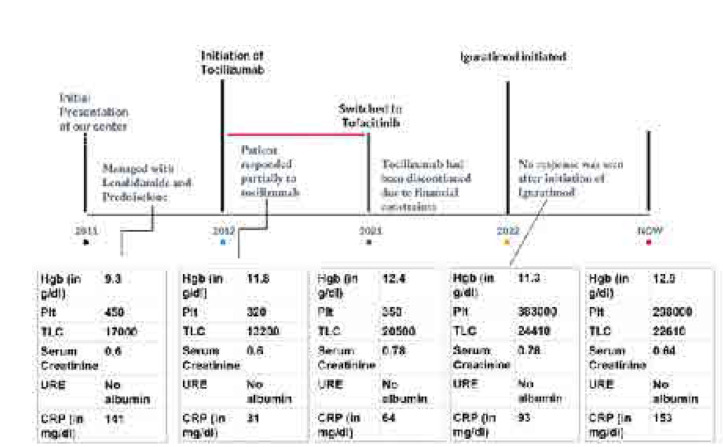
**(left).** Chronological timeline of clinical course for patient 3.

A 26-year-old gentleman presented to us at 13 years of age with a constellation of recurrent fever associated with an urticarial rash from 1 year of age, manifesting with diverse organ damage. Domains involved included ocular inflammation in the form of chronic anterior uveitis with retinal vasculitis. The major clue to an early diagnosis in this patient was the early subacute onset of bilateral sensorineural hearing loss with evidence of chronic aseptic meningitis with papilledema. His disease course had sporadic and recurrent episodes of inflammatory lower back pain and peripheral arthritis associated with persistently raised inflammatory markers despite immunosuppression (**Figure 3**). During his prolonged disease course, he failed several conventional synthetic DMARDs (**Figure 4**) and required long-term steroids for symptom control. Although he had a partial response to Tocilizumab, we could not continue it due to financial constraints. His whole exome sequencing revealed a heterozygous mutation in exon 3 of the *NLRP3* gene (variant of unknown significance).

**Table 2.** Improvement in domains of organ involvement and chronic inflammation (P2).

**Lab investigations**	**Pre-treatment**	**Latest follow up**
**Haemoglobin (g/dl)**	8	12.2
**TLC (cells/uL)**	17070	10400
**Platelets (cells/uL)**	656000	400000
**ESR (mm/hr)**	74	26
**CRP (mg/dL)**	33	3
**Slit lamp examination**	Bilateral anterior uveitis with no complications	No activity without topical prednisolone

**Table 3.** Summary of patient characteristics with domains of involvement.

**P. No.**	**Episodic Fever**	**Rash**	**Neurological**	**MSK**	**Eye**	**Hearing loss**	**Growth retardation**
**1.**	++	+	+++	+++	+	++	++ (pretreatment)
**2.**	+	++	-	++	+	-	+
**3.**	++	++	+++	++(Sacroiliitis)	+	+++	+++

He continues to need systemic steroids to control the inflammation and yet he continues to suffer from complex partial seizures, frequent flares of arthritis, and urticarial rashes. We are awaiting access to Anakinra or Tocilizumab for this patient.

Written informed consent was taken from all three families of the patients prior to publishing.

## DISCUSSION OF SIMILAR PUBLISHED CASES

Our cases seek to highlight the myriad manifestations and severity of *NLRP*3-AID in the Indian population, and we report a novel presentation of this disease in P3 in the form of Grade 4 sacroiliitis. The first description of *NLRP*3-associated autoinflammatory diseases draws several similarities to our present case series where three children presented with a pattern of episodic skin rash, fever, and chronic aseptic meningitis associated with intellectual disability.^5^ Twenty years prior, dermatologists had identified an autosomal dominant disease with urticaria, deafness, and amyloidosis.^4^

Cryopyrin-associated periodic syndromes (CAPS) encompass a range of systemic autoinflammatory disorders with varying clinical severity, stemming from a gain of function mutations in the CIAS1 gene.^3^ The gene is now identified to be *NLRP*3, which was recognised as a pivotal protein complex for caspase-1 activation and subsequent IL-1 activation and secretion.^6^

The latest nomenclature now identifies 3 syndromes in this phenotype. Recently, two new syndromes have been described associated with *NLRP3*, namely, Deafness Autosomal dominant 34 (DFN 34) with exclusive cochlear inflammation and Keratitis fugax hereditaria (KFH) with exclusive anterior uveitis.^2^

Neonatal onset of cutaneous urticarial rashes with no pruritus, angioedema, and an absence of response to antihistamines defines inflammation of the skin. In contrast to classical urticarial rash where mast cells predominate, perivascular neutrophilic infiltrate can be found on skin biopsy with no sign of vasculitis.

Neurological involvement can take several forms, ranging from chronic aseptic meningitis, headache and seizures. This chronic inflammation culminates in cognitive dysfunction which is modifiable with early diagnosis and appropriate treatment.^7^ Sensorineural hearing loss is often profound and contributes significantly to the morbidity and cognitive impairment associated with the disease.^8^ The hearing loss is attributed to inflammation of the cochlea demonstrable with a post-contrast enhancement of the cochlea.

The joint involvement in CINCA-NOMID syndrome varies widely among patients, from recurring episodes of joint pain and mild arthritis to severe joint deformities, often affecting the knees. Bone involvement is manifested by hypertrophic arthropathy as seen in 2 of our patients along with clubbing of digits with no pulmonary involvement. Histopathology of bony hypertrophy shows a disorganised endochondral ossification with ballooning of chondrocytes and mild nuclear irregularity. P1 and P3 also had clubbing attributable to the increased VEGF and PDGF as a result of *NLRP*3 activation.^9^

P3 presented with sacroiliitis which has been a well-established complication of another closely related autoinflammatory disease - familial Mediterranean fever but has not been reported in *NLRP3-associated* AID. We report this manifestation for the first time in our case series.

Cryopyrinopathies are characterised by a cytokine milieu rich in IL-1β and IL-18. IL-1β, serves to enhance IL-2R on T cells and also stabilising intracellular IL-2R mRNA resulting in florid T cell proliferation, while IL-18 also serves to enhance B cell proliferation and differentiation into plasmablasts.^10–12^ Further, IL-1β in conjunction with IL-6 they upregulate the expression of adapter molecules like STAT-3 and transcription factors IRF-4 and ROR-γt in T cells; thus, skewing the adaptive T cell responses from Treg phenotype to proinflammatory, pathogenic Th17 phenotype.^13^ This pathogenic skew in adaptive T cell responses and consequent pathogenic IL-17 enriched state appears to be an intriguing pathophysiologic basis of articular manifestations akin to spondylarthritis observed in *NLRP*-3 associated auto-inflammatory disease. The therapeutic vantage point of this hypothesis needs further exploration^14^

Eye manifestations can range from recurrent conjunctivitis, uveitis and papilledema. These manifestations are fairly common and have been reported in various other cohorts with varying frequency.^15^ Resolution of papilledema on optical coherence tomography can be used to objectively assess response to therapy on follow up as well as with P3. Apart from the strikingly characteristic presentations mentioned above, most children have growth retardation.^16^

The majority of these patients (50–60%) have heterozygous gain-of-function missense mutations in the exon 3 of CIAS1 gene, which codes for NACHT domain of *NLRP*-3 protein.^17,18^ These mutations affect the autoinhibitory interactions between C-terminal leucine rich repeat, sensing motif and intermediate NACHT motif, resulting in un-checked inflammasome activation. All 3 patients in our case series were identified to have a mutant heterozygous allele in exon 3 of *NLRP*3 ^2^ The family history of all 3 patients were unyielding as significant number of cases result from de-novo somatic mutations resulting in varied degrees of mosaicism and consequent heterogeneity in age of onset and severity of the disease. A clear genotype- phenotype correlation could not be established owing to the paucity of data (**Table 4**).

**Table 4.** Genetic reports of our 3 children.

**Diagnosis**	**Gene (Transcript)**	**Location**	**Variant**	**Zygosity**	**Classification**	**Inheritance**	**Family History**
**P1: NOMID**	NLRP 3 (+) ENST00000336119.8	Exon 3	c.1985T>C, p.662M>T	Heterozygous	Pathogenic	Autosomal Dominant	None
**P2: NOMID**	NLRP 3 (+) ENST00000336119.3	Exon 3	c.1697G>T, p.Gly566Val	Heterozygous	Likely Pathogenic	Autosomal Dominant	None
**P3: MWS**	NLRP 3 (+) ENST00000336119.3	Exon 3	c.1706A>C, p.Glu569Ala	Heterozygous	Variant of Unknown Significance	Autosomal Dominant	None

All our patients were labelled systemic JIA and had an inadequate response to therapy. Differential diagnoses considered included CRMO, Enchondroma and bone dysplasia to attribute for the abnormal hypertrophy of the bone.

Secondary AA amyloidosis is a life-threatening complication seen in several systemic autoinflammatory disorders like FMF, TNF-receptor periodic associated syndrome, and Muckle-Wells syndrome, and has been sporadically reported in CINCA-NOMID syndrome cases.^19^ This is likely due to the comparatively lower life expectancy of CINCA-NOMID patients when compared to those with other systemic autoinflammatory diseases.

In terms of treatment, first-line therapy with the most available evidence is the blockade of the IL-1β pathway with Anakinra, rilonacept and canakinumab.^7,20^ As is evident from our three cases, the first benefitted the most from initiation of Anakinra and other therapeutic alternatives merely act as a bridge to gain access to IL-1β blockade. Delay in diagnosis and treatment may lead to long term complications and inadequate control of inflammation, eventually leading to amyloidosis, growth retardation, adverse effects of glucocorticoids and alternative immunosuppression. Although P3 suffers from long term sequelae of persistent inflammation, the lack of development of amyloidosis stands as an optimistic testament to the efficacy of these alternative immunosuppression. Thalidomide and lenalidomide may be reasonable, albeit less effective alternatives in countries with poor access to IL-1 inhibition, limited by its toxicity.^21^

## CONCLUSION

A low clinical threshold for genetic testing in any child who displays features of autoinflammation in combination with an urticarial rash, musculoskeletal manifestations, hearing loss and chronic aseptic meningitis with macrocephaly must be considered. We also present a unique case of *NLRP*-3 AID with sacroiliitis as a prominent feature. These syndromes show a robust response to IL-1 blockade owing to the cytokine milieu that is inherent to the disease state. Access to anakinra in resource limited settings, paves the way for alternatives like colchicine and thalidomide. However, our cases demonstrate that there is an urgent need to provide access to these medications in rare diseases.

## FUNDING INFORMATION

No Source of Funding is applicable for this study.

## ETHICAL STATEMENT

The work described has been carried out in accordance with The Code of Ethics of the World Medical Association (Declaration of Helsinki) for experiments involving humans.

## INFORMED CONSENT

Informed consent was taken for all applicable patients in the manuscript.

## DECLARATION OF CONFLICT OF INTEREST

All authors report no conflicts of interest.

## AUTHOR CONTRIBUTIONS

Suma Balan, Mithun CB: conceived, designed and revised the manuscript.

Sumanth Madan, Spoorthy Raj: Drafting the paper, literature review.

Joslyn Mary Thattil: manuscript review, literature review.

Sudeep Rath, Ashvin Pillai: Data collection and illustrations.

All authors reviewed the results and approved the final version of the manuscript.
